# The Polycystic Ovary Syndrome and the Metabolic Syndrome: A Possible Chronobiotic-Cytoprotective Adjuvant Therapy

**DOI:** 10.1155/2018/1349868

**Published:** 2018-07-25

**Authors:** Eduardo Spinedi, Daniel P. Cardinali

**Affiliations:** ^1^Centre for Experimental and Applied Endocrinology (CENEXA, UNLP-CONICET-FCM), CEAS-CICPBA, La Plata Medical School, La Plata, Argentina; ^2^BIOMED-UCA-CONICET and Department of Teaching and Research, Faculty of Medical Sciences, Pontificia Universidad Católica Argentina, Buenos Aires, Argentina

## Abstract

Polycystic ovary syndrome is a highly frequent reproductive-endocrine disorder affecting up to 8–10% of women worldwide at reproductive age. Although its etiology is not fully understood, evidence suggests that insulin resistance, with or without compensatory hyperinsulinemia, and hyperandrogenism are very common features of the polycystic ovary syndrome phenotype. Dysfunctional white adipose tissue has been identified as a major contributing factor for insulin resistance in polycystic ovary syndrome. Environmental (e.g., chronodisruption) and genetic/epigenetic factors may also play relevant roles in syndrome development. Overweight and/or obesity are very common in women with polycystic ovary syndrome, thus suggesting that some polycystic ovary syndrome and metabolic syndrome female phenotypes share common characteristics. Sleep disturbances have been reported to double in women with PCOS and obstructive sleep apnea is a common feature in polycystic ovary syndrome patients. Maturation of the luteinizing hormone-releasing hormone secretion pattern in girls in puberty is closely related to changes in the sleep-wake cycle and could have relevance in the pathogenesis of polycystic ovary syndrome. This review article focuses on two main issues in the polycystic ovary syndrome-metabolic syndrome phenotype development: (a) the impact of androgen excess on white adipose tissue function and (b) the possible efficacy of adjuvant melatonin therapy to improve the chronobiologic profile in polycystic ovary syndrome-metabolic syndrome individuals. Genetic variants in melatonin receptor have been linked to increased risk of developing polycystic ovary syndrome, to impairments in insulin secretion, and to increased fasting glucose levels. Melatonin therapy may protect against several metabolic syndrome comorbidities in polycystic ovary syndrome and could be applied from the initial phases of patients' treatment.

## 1. Introduction

Polycystic ovary syndrome (PCOS) is a highly frequent reproductive-endocrine disorder detected in up to 8–10% of women at reproductive age worldwide. Due to its heterogeneous nature, different criteria have been established in the course of several years in order to lead to a more precise PCOS diagnosis [1–5]. Presently, accepted criteria indicate that PCOS patients are characterized by oligomenorrhea or amenorrhea, hyperandrogenism, and/or hyperandrogenemia, with enlarged ovary volume full of cystic ultrasound images [1].

Although the etiology of PCOS is not fully understood, evidence suggests that insulin resistance (IR), with or without compensatory hyperinsulinemia, contributes to inhibit liver sex hormone-binding globulin (SHBG) production and to stimulate ovarian/adrenal androgen secretion. Dysfunctional white adipose tissue (WAT) has been identified as a major contributing factor for IR in PCOS. Environmental and genetic/epigenetic factors may also play relevant roles in PCOS development [6, 7].

The dysmetabolic aspect of PCOS has recently gained much attention. Several features of metabolic disturbances, particularly IR and hyperinsulinemia, have been observed in most women with PCOS [8]. Therefore, overweight and/or obesity (specifically, enlarged visceral fat) are very common in women with PCOS, thus indicating that some PCOS and metabolic syndrome (MS) female phenotypes share common characteristics [9].

MS is a cluster of endocrine-metabolic dysfunctions including IR, overweight/central obesity, dyslipidemia, hypertension, and high risk of cardiovascular disease [10]. In this regard, studies confirm that MS is more common among PCOS patients due to the higher prevalence of IR and hyperadiposity (visceral) in these women [11, 12]. Moreover, PCOS patients have an elevated risk of MS [12], indicating that WAT dysfunction is a highly prevalent and common feature in the PCOS-MS phenotype [4, 13].

Although not yet deeply investigated, sleep disturbances have been reported to double in women with PCOS. Indeed, PCOS patients displayed increased difficulty in falling to sleep and staying asleep [14], with excessive daytime somnolence being higher in sleep-disordered than in not sleep-disordered PCOS women [15]. In this regard, obstructive sleep apnea is a common feature in adult [16] and adolescent [17] PCOS patients suffering multiple comorbidities, such as IR and hyperadiposity. Significant chronodisruption occurs in patients suffering from obstructive sleep apnea as evidenced by a disrupted melatonin circadian rhythm [18]. A clear relationship between sleep disturbances and reproductive dysfunction has also been noticed in non-PCOS women: those sleeping less than 6 h daily have shorter or longer menstrual cycles [19].

Experiments using neonatally androgenized female rats (the Barraclough PCOS model) indicated an altered pattern of hypothalamic luteinizing hormone-releasing hormone (LHRH) and pituitary LH/FSH contents directly related to changes in the pulsatile pattern of LH/FSH release [20]. In this regard, maturation of the LHRH secretion pattern in girls across puberty has been reported to be closely related to changes in the sleep-wake cycle and is suggested to be relevant in the pathogenesis of PCOS [21].

Overall metabolic-reproductive-cardiovascular risk associated with PCOS urges for a more broadly specific therapeutic approach in medical care of PCOS patients. This short review article focuses on two main issues in PCOS-MS phenotype development: (a) the impact of androgen excess on WAT function and (b) a possible adjuvant melatonin therapy to improve the chronobiologic profile in PCOS individuals.

## 2. Cooperative Effect of Androgens on White Adipose Tissue Dysfunction

Adipose tissue mass and distribution pattern display clear gender/sex dimorphism [22, 23]. Whereas men have greater predisposition to accumulate visceral adipose tissue (VAT) (known as android distribution) [23], women accumulate WAT in the gluteofemoral position (known as gynoid distribution) [22]. VAT mass expansion is associated with a higher risk of type 2 diabetes mellitus and cardiovascular disease [24]. The relationship between blood androgen levels and WAT function in women seems to be more complex. It is accepted that androgen excess is associated with central obesity, although some studies deny this assumption. PCOS phenotypes often have hyperandrogenemia associated with IR and accumulate WAT mass [25, 26]. Testosterone is able to directly induce IR in adipocytes [27], in part by decreasing cellular glucose uptake [28].

Studies performed on the preadipocyte cell line 3T3-L1 and on multipotent cell lines (C3H10T1/2) indicate that testosterone inhibits cell proliferation and differentiation to mature adipocytes [29–31]. A similar inhibitory testosterone effect is found in human adipocyte precursor cells (APCs) from distinct WAT pads. It is possible that the antiadipogenic action of androgens is due to the inhibiting activity of PPAR-*γ*2 and C/EBP-*α* functions; interestingly, induction of APCs to differentiate results in increased AR expression [32]. Moreover, it is widely accepted that inhibition of the adipogenic process results in hypertrophic WAT mass expansion due to enlargement of the local adipocyte size [33]. Thus, in spite of its inhibitory effect on adipogenesis, it seems feasible that testosterone could induce WAT (VAT) pad mass expansion by increasing the cell size (hypertrophic pad mass expansion). Most studies of androgen effects on the adipogenic process have focused on the terminal phase of the process, then it remains to be determined whether testosterone acts on other stages of adipogenesis (e.g., by influencing APC number and competency).

It must be taken into account that hypertrophy expansion of WAT mass, as it occurs in an androgen excess endogenous milieu, is due to the large size of local adipocytes that release large amounts of proinflammatory adipocytokines [such as leptina (LEP), resistin, tumor necrosis factor alpha (TNF*α*), plasminogen activator inhibitor type-1 (PAI-1), interleukin-1 (IL-1), and IL-6, among others]. These substances are highly capable of diminishing tissue insulin sensitivity and promoting oxidative stress (OS). Moreover, hypertrophic WAT cells did also release few amounts of adiponectin (ADIPOQ), an insulin-sensitizing adipokine, thus worsening local (white adipocyte) and peripheral tissue insulin sensitivity.

**Figure 1:**
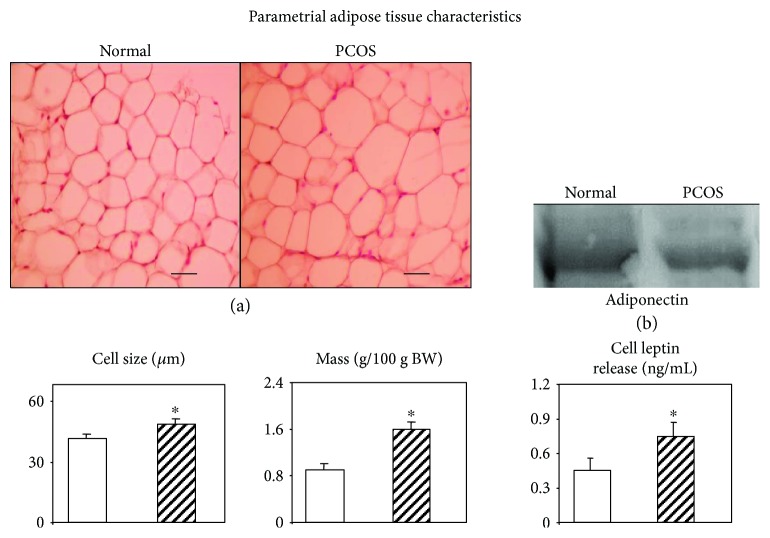
White adiposity characteristics in normal and PCOS adult rats. Representative images of parametrial adipose tissue pads stained with hematoxylin and eosin (a). Lower 3-diagram panel showing data from parametrial pad adipocyte (in left-right order) size, mass, and *in vitro* leptin secretion. Finally, parametrial pad adiponectin protein content (Western blot) (b). Magnification ×400; scale bars: 50 *μ*m. ^∗^*P* < 0.05 versus respective normal-group values (adapted from Alzamendi et al. [34]).

Interestingly, studies in the rodent neonatal androgenization model of PCOS clearly showed that early transient testosterone excess in female rats resulted in an adult phenotype characterized by several endocrine-metabolic dysfunctions [34]. Indeed, these rats showed altered WAT (parametrial tissue-pad) functionality, such as enlarged pad mass, replete of large (insulin-resistant) adipocytes and containing very low ADIPOQ protein [34]. Moreover, their isolated WAT adipocytes did release excess of LEP and resulted to be highly resistant to insulin-stimulated LEP secretion [34]. These dysfunctional PCOS rats were peripherally characterized by increased levels of LEP, PAI-1, and nonesterified fatty acids and decreased ADIPOQ concentration [34]. Their responses to an ip glucose tolerance test indicated (by analyzing the area under the curve values) that although they were still fully able to manage glycemias (normal glucose tolerance), this occurred at the expenses of compensatory hyperinsulinemias, whereas adiponectinemias remained very low [34]. Some of these altered functions are shown in Figure 1. The alterations indicate that a phenotype shifted towards an overall state of IR and inflammation. Relevantly, neonatal treatment with flutamide, a nonsteroidal antagonist of the androgen receptor, induced a decrease in the peripheral levels of leptin [35]. Collectively, these data indicate that early treatment with testosterone increases susceptibility to MS [36] development and, conversely, that flutamide treatment improves this condition. These observations strongly support that testosterone exerts specific receptor-mediated effects [37], the androgen receptor being expressed in both white adipocytes and APCs [38]. However, the level of androgen receptor expression differs among WAT depots. VAT has higher receptor expression levels than subcutaneous fat deposits [32, 38, 39], which could explain in part the differential actions of testosterone on different adipose tissue depots.

**Figure 2:**
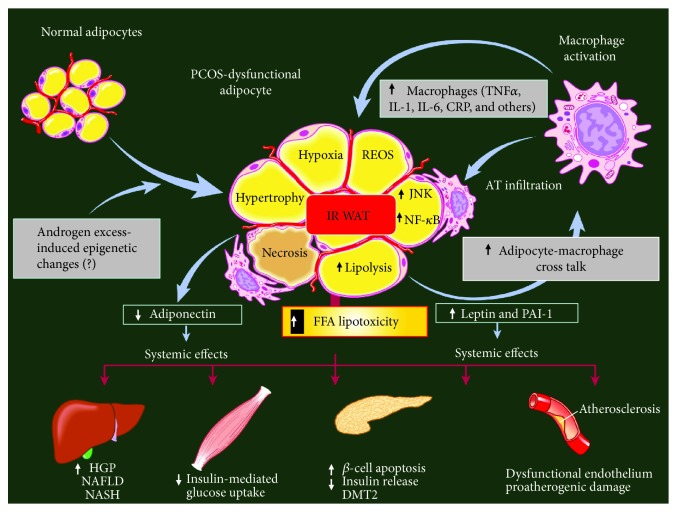
White adipose tissue (WAT) and inflammation: endocrine-metabolic consequences. A combination of genetic background and endogenous androgen excess could induce WAT mass hypertrophic expansion associated with macrophage infiltration, leading to an abnormal pattern of adipokine secretion. Enhanced WAT-derived leptin release, in turn, impairs tissue sensitivity to insulin (insulin resistance (IR)). Prolonged hyperleptinemia could induce long-form leptin receptor (ObRb) downregulation, namely, at the pancreatic (*β*- and *α*-cell) level, thus impairing its negative feedback mechanism on insulin (and glucagon) secretion; moreover, increased release of proinflammatory signals (TNF, IL-1, IL-6, and C-reactive protein (CRP), among others) worsens several functions. In fact, overall WAT dysfunction promotes multiple endocrine-metabolic dysfunctions, such as generalized IR, enhanced reticulum endoplasmic oxidative stress (REOS), enhanced lipolytic activity, cell hypoxia, and apoptosis. These alterations, in turn, affect multiple peripheral organs, namely, liver, muscle, endocrine pancreas, and endothelium functions. FFA: free fatty acid; JUNK: Janus kinase; NF-*κ*B: nuclear factor-*κ*B; HGP: hepatic glucose production; NAFLD: nonalcoholic fatty liver disease; NASH: nonalcoholic steatohepatitis; DMT2: diabetes mellitus type 2 (adapted from Pagano et al. [108]).

Altered lipolysis/lipogenesis balance contributes to the increase in lipid storage in adipose cells and therefore to the development of unhealthy (hypertrophic) VAT mass expansion. As mentioned above, androgen excess is per se a clear inducing factor of white adipocyte hypertrophy, and these cells overproduce (enhanced synthesis and secretion) proinflammatory adipokines (including angiotensinogen and free fatty acids), resulting in a local inflammatory state. Large adipocytes are indeed insulin-resistant (IR) and characterized by high cell reticulum endoplasmic oxidative stress (REOS) content, enhanced lipolytic activity, cell hypoxia, and apoptosis. Moreover, large IR adipocytes are not well recognized by the immune system, and, as a consequence, macrophages react against to and infiltrate dysfunctional WAT, thus worsening the inflammatory state. These alterations in PCOS women, in turn, affect multiple organ functions, namely, at liver [40], muscle [41], endocrine pancreas [42], and endothelium [43] levels, thus compromising cardiovascular function (hypertension and atherogenesis) [44] (Figure 2).

**Figure 3:**
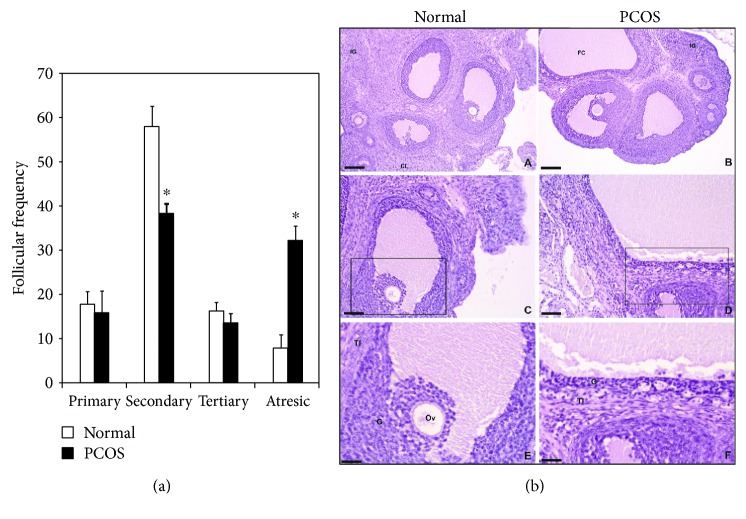
Follicular developmental stage of the ovaries obtained from normal and PCOS adult rats (a). Values are expressed in percentages. ^∗^*P* < 0.05 versus normal-group values. Representative images (b) of the ovaries from normal (A, C, and E) and PCOS (B, D, and F) adult rats showing ovarian structures at different magnifications: (A) and (B): 10x (bars: 100 *μ*m); (C) and (D): 20x (scale bars: 50 *μ*m); (E) and (F): 40x (scale bars: 25 *μ*m). CL: corpus luteum; IG: interstitial glands; FC: follicular cyst; G: granulosa; TI: theca interna; Ov: ovocyte (adapted from Ongaro et al. [45]).

Regarding ovarian function, recent studies from one of our laboratories using the PCOS rat phenotype indicated a clear ovary dysfunctional folliculogenesis [45] as indicated by the number of secondary and atretic stage follicles; indeed, a 3-fold lower number and a 5-fold higher number, respectively, were noticed in the PCOS rat phenotype than in the normal rat (Figure 3(a)). Moreover, ovary images in normal animals fully correspond with expected characteristics (e.g., antral cavity, corpus luteum, oocyte, granulosa cells, and internal and external theca cells) (Figure 3(b)), whereas those from PCOS rats displayed dysfunctional characteristics compatible with a large antral cavity in a cystic follicle and showed decreased granulosa and theca (internal and external) cell layers (Figure 3(b)). This misprogramming in carbohydrate metabolism and dyslipidemia (e.g., a prediabetic state) as well as the inflammatory state indicate that the PCOS rat phenotype is highly prone to developing cardiovascular disease and reproductive dysfunction (abnormal folliculogenesis). Therefore, subfertility/infertility and/or poor pregnancy outcome (early abortion and/or preeclampsia) could ensue.

## 3. Chronobiology in PCOS

As mentioned, some PCOS phenotypes carry a significant risk for metabolic disturbances including MS, prediabetes, and type 2 diabetes and an intrinsic prooxidant state resulting from imbalance between excessive oxidant production in the presence of limited antioxidant defence. MS comprises many risk factors for cardiovascular disease including hyperinsulinemia, glucose intolerance, dyslipidemia, hyperadiposity/obesity, and elevated blood pressure. MS prevalence ranges from 15 to 30% depending on the world region considered [46, 47]. A 1.5- to 2.5-fold increase in cardiovascular mortality occurs when MS is present, representing one of the major public health problems at this time.

In the last decade, the understanding of the cellular and molecular events that contribute to MS development has increased considerably. One basic function apparently heavily influenced by obesity and metabolic disease is the internal timing system [48–50]. The correlation between increased occurrence of obesity and the ubiquity of modern social habits, such as light at night, unusual meal times, and irregular sleep/wake schedules, all encompassed by a “24/7” lifestyle, strongly suggests that impairment of sleep and the circadian system is involved in the etiology of MS. Several clinical surveys have shown increased prevalence of MS in night-shift workers, indicating that artificial lighting may contribute to increased prevalence of metabolic disorders [51–55].

Because melatonin, as a chronobiotic/cytoprotective agent, has a special place in prevention and treatment of MS [52–54], its possible therapeutic utility in PCOS has been considered. Low levels of melatonin at night have been linked to metabolic abnormalities such as insulin resistance and type 2 diabetes mellitus. Moreover, the suppression of nocturnal melatonin by light exposure at night has been associated with several pathologies comprising MS [56–58].

Melatonin is measurable in human preovulatory follicular fluid and may play a role in regulating ovarian steroidogenesis, folliculogenesis, and oocyte maturation [59]. Melatonin can protect follicles against oxidative stress and may rescue follicles from atresia, thereby promoting correct follicular maturation and, ultimately, ovulation [60].

Table 1 summarizes results supporting a therapeutic role of melatonin in PCOS. In rats, the reduction of circulating melatonin levels after pinealectomy induces the development of some characteristics of PCOS. Published data indicate that the direct effect of melatonin on follicular steroid production is complex and may depend on the cell type (theca cell or granulose cell), duration of treatment (acute or long-term response), experimental model (cell culture or follicle culture), species, and dose [61]. Melatonin may directly suppress follicular (thecal) steroidogenesis at an early stage in the steroid synthesis pathway by blocking the expression of steroidogenic acute regulatory protein, which facilitates translocation of cholesterol across the intermembrane space into the inner membrane to be cleaved into pregnenolone. Treatment of rats with melatonin can reduce obesity, type 2 diabetes, and hepatic steatosis [62, 63], and in several animal models of hyperadiposity melatonin injection normalized most observed alterations and corrected the altered biochemical proinflammatory profile.

A significantly higher excretion of 6-sulfatoxymelatonin, the major excretory metabolite of melatonin, was reported in patients with PCOS [64–66]. Other studies later described higher serum melatonin concentrations associated with ovarian intrafollicular deficiency of melatonin in patients with PCOS [67, 68]. Regardless of that, the intrafollicular melatonin concentrations are lower in PCOS than in controls [61]. The high circulating levels of melatonin in PCOS may be a feedback response to the deficient levels of melatonin in the ovary [61]. High levels of melatonin in the follicular fluid is essential for follicle growth, ovulation, and oocyte quality, whereas reduced follicular melatonin concentrations may be responsible for anovulation and poor oocyte quality in PCOS.

Melatonin communicates nightly timing cues through activation of two G protein-coupled receptors, that is, melatonin receptor 1 (MT1) and melatonin receptor 2 (MT2) [68]. Both MT1 and MT2 have been shown to activate several signalling pathways, most notably the Gi/cAMP and Gq/phospholipase C/Ca^2+^ pathways. These receptors are expressed in many different peripheral tissues such as the ovary and modulate multiple aspects of human physiology [69].

Genome-wide association studies have shown that polymorphisms in the genes encoding human melatonin receptors (MTNR1A and MTNR1B) are involved in the pathogenesis of type 2 diabetes mellitus [70–76]. By resequencing the coding region of the MTNR1B gene coding for the MT2 receptor, variants have been identified and functionally characterized. Corresponding mutants with impaired receptor signalling are strongly associated with diabetic risk, indicating that loss of melatonin receptor function is positively associated with disease risk.

Variants in MTNR1B (MT2) have been linked to impairments in both insulin secretion and increased fasting glucose levels, and variants in MTNR1A (MT1) have been shown to be associated with increased risk of developing PCOS [77–80]. Polymorphisms rs2119882 in the MTNR1A gene and rs10830963 in the MTNR1B gene were proposed to have a common causative role in the pathogenesis of PCOS. However, in a recent study to investigate whether an association exists between these two single-nucleotide polymorphism variants and PCOS, an association was detected only between rs2119882 in the MTNR1A gene and PCOS [77]. Collectively, genetic data provide a basis for further studies of the MTNR gene in the etiology of PCOS.

Melatonin may directly affect ovarian function: it is concentrated in human ovarian follicles relative to the level in plasma, and it alters granulosa cell steroidogenesis and follicular function in humans [60]. However, only a few studies have been published on melatonin potential as a therapeutic agent in humans in the PCOS.

Two of them relate to improvement of in vitro fertilization of patients with PCOS. The supplementation of in vitro culture medium with melatonin improved in vitro fertilization outcome in PCOS [81] while melatonin and myo-inositol enhanced, synergistically, oocyte and embryo quality and improved in vitro fertilization of patients with PCOS [82]. These findings suggest that the addition of melatonin to in vitro fertilization media may improve the cytoplasmic maturation of immature oocytes.

In an open-label study including 40 normal-weight women with PCOS, ultrasound pelvic examinations, hirsutism score evaluation, hormone profile assays, oral glucose tolerance test, and lipid profile at baseline and after a 6-month administration of 2 mg fast release melatonin po daily at bedtime were recorded [83]. Melatonin treatment significantly decreased serum androgen and 17*α*-hydroxyprogesterone levels and augmented serum FSH and anti-Mullerian hormone serum levels. Almost 95% of participants experienced an amelioration of menstrual cycle disruption. No significant changes occurred in glucoinsulinemic and lipid parameters after treatment except a significant decrease in low-density lipoprotein cholesterol.

Treatment of preinvasive endometrial cancer in women with PCOS using melatonin in combination with female sexual hormones and antidiabetic, antidopaminergic, and antiserotoninergic therapy favorably influenced female sexual hormone profile and lipid metabolism and caused restoration of normal endometrium [84]. Indeed, melatonin treatment ameliorated oxidative stress and inflammatory parameters of obese women [85] and reduced fat mass and increased lean mass in postmenopausal women [86].

Therefore, the data in Table 1 agree with many studies now supporting the beneficial role of melatonin in patients with MS. Melatonin treatment ameliorates MS in obese patients [87, 88] as well as in bipolar and schizophrenic patients after treatment with second-generation antipsychotics [89–91]. Melatonin administration normalizes MS in elderly hypertensive patients [92] and improves enzyme profile in patients with alcoholic liver steatosis [88, 89]. Using melatonin and zinc acetate, when employed alone or in combination with metformin, improved glycemic control in type 2 diabetic patients [93], and an inverse relationship between urinary 6-sulfatoxymelatonin excretion and insulin levels versus insulin resistance was reported in healthy women in the Nurses' Health Study cohort [94]. It must be noted however that there are results that deny the capacity of melatonin to improve glucose tolerance and to reduce insulin resistance in humans. Melatonin administration decreased glucose tolerance, already in nondiabetic young individuals [95–97]. Although the results summarized in Table 1 suggest that melatonin therapy may be beneficial for patients with PCOS, more studies are obviously needed to evaluate an appropriate time/duration of treatment/dose relationship for administration of melatonin.

## 4. Concluding Remarks

Many metabolic-reproductive alterations associated with PCOS are closely dependent on WAT dysfunction, particularly at the VAT pad level. However, the increase in VAT pad mass per se is not an unequivocal indication of VAT dysfunction, whereas development of enlarged local adipocytes is indeed a key factor. Androgen excess is able to induce an imbalance between white adipocyte hypertrophy and hyperplasia, towards enlarged (IR) adipocytes and consequent VAT dysfunction and inflammation. Many factors regulate normal VAT mass expansion. In this review, we ponder the influence of testosterone, a sex steroid hormone in excess (hyperandrogenemia and/or hyperandrogenism) in PCOS phenotypes. Factors modulating adipogenesis/inflammation could become new therapeutic targets to counteract endocrine-metabolic-reproductive dysfunction, favoring healthy WAT mass expansion and counteracting hypertrophic adiposity-associated dysfunctions.

According to the Rotterdam ESHRE/ASRM-Sponsored PCOS Consensus Workshop Group, it was concluded that PCOS is a syndrome of ovarian dysfunction along with the cardinal feature hyperandrogenism and polycystic ovary (PCO) morphology [1]. However, because PCOS remains a syndrome, not a single diagnostic criterion is sufficient for clinical diagnosis. There are many other clinical manifestations that may be included, such as menstrual irregularities, signs of androgen excess, obesity, insulin resistance, elevated serum levels of LH, increased risk of type 2 diabetes [98], and cardiovascular events [99]. Clinical management of PCOS should include rigorous lifestyle modifications, insulin therapy, and drug treatments that promote insulin sensitization (such as metformin) and insulin secretion (such as glibenclamide), dipeptidyl peptidase-4 inhibitors, sodium glucose cotransporter 2 inhibitors [100], and antihyperlipidemic therapy [101]. In general, these approaches are designed to manage symptoms of insulin resistance/*β*-cell dysfunction and dyslipidemia and are used either alone or in combination. Drug therapies are expensive worldwide and in some cases have been associated with adverse secondary events including pancreatitis, hypoglycemia, and osteoporosis [102, 103]. Therefore, a need remains for new and cost-effective pharmacotherapies for diabetes presenting limited additional health risks.

Although overweight/obesity is preventable, its prevalence is continuously increasing worldwide, and because it is frequently associated with other cardiovascular risk factors and high mortality, obesity has become an important public health problem and a heavy socioeconomic burden for society as a whole. Environmental factors, hormone excess (e.g., androgen), or stressors of so-called contemporary “24/7” societies have pronounced effects on metabolism producing circadian clock disruption. Further, people whose work involves irregular time schedules and forced exposure to bright light at night (night/shift workers) show significant disruptions in sleep architecture and increased prevalence of MS. These lines of evidence indicate that the body's system fails to adjust properly to environmental and/or stressor changes disrupting overall metabolic homeostasis.

**Figure 4:**
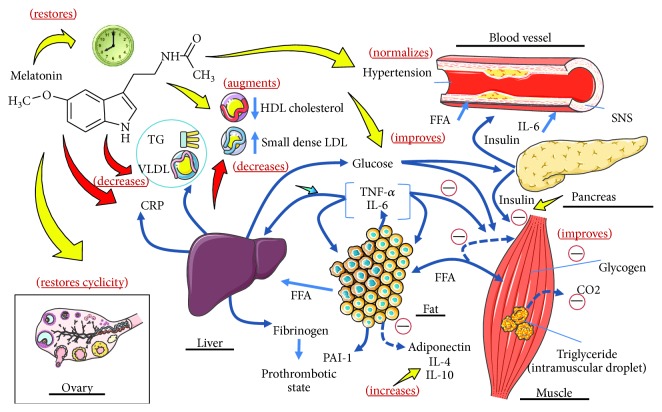
Effect of melatonin in PCOS associated with metabolic syndrome. Melatonin normalizes high blood pressure (BP) and circulating indexes of inflammation. It also improves insulin sensitivity and restores disrupted circadian rhythms. Melatonin directly affects ovarian function: it is concentrated in human ovarian follicles in relation to the level in plasma, and it improves granulosa cell steroidogenesis and follicular function in humans (adapted from Reiter et al. [61]).

Melatonin may provide an innovative strategy in PCOS by combining its chronobiotic effect on circadian rhythm with cytoprotective properties. Indeed, melatonin protects against several MS comorbidities in PCOS, such as diabetes and concomitant oxy-radical mediated damage, inflammation, microvascular disease, atherothrombotic risk, and ovary dysfunction. Melatonin may therefore have a place from the initial phases of PCOS treatment. Its high safety profile and reduced toxicity distinguishes it from many pharmaceutical agents used in PCOS patients (Figure 4). In conclusion, an appropriately classical pharmacological treatment combined with melatonin should be considered in PCOS individuals to restore endocrine-metabolic and reproductive functions.

## Figures and Tables

**Table 1:** Relevance of melatonin in PCOS.

Observation	Reference/s
Significantly higher secretion of melatonin in PCOS women	[64–68]
Family association study between melatonin receptor gene polymorphisms and PCOS	[77–79]
Supplementation of in vitro culture medium with melatonin improved IVF outcome in PCOS	[80]
Melatonin and myo-inositol enhanced, synergistically, oocyte, and embryo quality and improved in vitro fertilization of patients with PCOS	[81]
Melatonin treatment restores menstrual cyclicity in women with PCOS	[82]
Treatment of preinvasive endometrial cancer in women with PCOS using female sexual hormones in combination with melatonin, antidiabetic, antidopaminergic, and antiserotonin therapy favorably influenced female sexual hormone profile and lipid metabolism and caused the restoration of normal endometrium	[83]
Melatonin treatment ameliorated oxidative stress and inflammatory parameters of obese women	[84]
Reduced fat mass and increased lean mass in response to 1 year of melatonin treatment in postmenopausal women	[85]
Protective effects of melatonin against metabolic and reproductive disturbances in rodent models of PCOS	[104–107]
